# High-Repetition-Rate Femtosecond Laser Processing of Acrylic Intra-Ocular Lenses

**DOI:** 10.3390/polym12010242

**Published:** 2020-01-20

**Authors:** Daniel Sola, Rafael Cases

**Affiliations:** 1 Institut für Fertigungstechnik, Technische Universität Dresden, 01069 Dresden, Germany; 2 Laboratorio de Óptica, Centro de Investigación en Óptica y Nanofísica, Universidad de Murcia, Campus Espinardo, 30100 Murcia, Spain; 3 Departamento de Física de la Materia Condensada, Universidad de Zaragoza, 50009 Zaragoza, Spain; cases@unizar.es

**Keywords:** laser processing, femtosecond pulses, ophthalmic polymers, Raman spectroscopy

## Abstract

The study of laser processing of acrylic intra-ocular lenses (IOL) by using femtosecond laser pulses delivered at high-repetition rate is presented in this work. An ultra-compact air-cooled femtosecond diode laser (HighQ2-SHG, Spectra-Physics) delivering 250 fs laser pulses at the fixed wavelength of 520 nm with a repetition rate of 63 MHz was used to process the samples. Laser inscription of linear periodic patterns on the surface and inside the acrylic substrates was studied as a function of the processing parameters as well as the optical absorption characteristics of the sample. Scanning Electron Microscopy (SEM) Energy Dispersive X-ray Spectroscopy (EDX), and micro-Raman Spectroscopy were used to evaluate the compositional and microstructural changes induced by the laser radiation in the processed areas. Diffractive characterization was used to assess 1st-order efficiency and the refractive index change.

## 1. Introduction

Polymeric materials have become well-known in recent decades and have experienced an outstanding development achieving unique properties that have allowed them to enter rapidly in almost all industrial, technological and biotechnological applications in semiconductor manufacturing and coatings, household appliances, automotive, electronics, aerospace, as well as in biomedicine, bioengineering, pharmaceutical and ophthalmology. It is worth noting their application like lab-on-chip devices, storage devices, optoelectronic and photovoltaic devices, micro-fluidic channels, orthopedic, dental, hard and soft tissue replacements, cardiovascular devices, drug delivery, and as contact and intraocular lenses [1,2,3,4,5,6,7,8,9,10,11,12]. In fact, polymers represent the largest class of materials used for biomedical applications. The reasons why they are the preferred material are resource efficiency and energy saving, easy and reliable processing capabilities for diverse styling and design. In addition, polymers usually have excellent bulk physical and chemical properties such as low surface energy, hydrophobicity, and high electrical resistance [1,2].

Among polymers, acrylic polymers have become one of the most popular materials due to its excellent properties, including optical transparency from the UV to the NIR spectral region, flexibility, elasticity, tunable mechanical properties, oxygen permeability, hydrophobicity, biocompatibility, biostability, durability and low cost [1,7,13]. This versatility has allowed acrylic polymers to be used as micro total analysis systems (μ-TAS), micro-electro-mechanical systems (MEMS), microfluidic channels, waveguides, as well as in numerous pharmaceutical and medical applications as neural implants or intraocular lenses [12,13,14,15,16,17,18,19,20,21].

Ultrafast Laser Inscription (ULI) has become a powerful and versatile technique for selective surface and bulk processing. In this technique, ultra-short laser pulses are tightly focused inside transparent materials inducing nonlinear absorption processes in the focal volume and leading to permanent weak local refractive index variations, formation of nano-voids, crystallization processes or even chemical transformations. This technique has been widely used to modify crystalline and glassy matrices as well as polymers to produce passive and active photonic devices, to create 2D/3D micro/nanostructures or to activate and functionalize the surface [15,16,17,18,19,20,22,23,24,25,26,27,28,29,30,31,32,33,34,35]. Nevertheless, despite laser ablation of polymers is a well stablished process for industrial applications the contribution of the main mechanisms which may take part in the laser-polymer interaction process, i.e., photo-chemical and photo-thermal decomposition processes, are not clearly solved and the discussion is still controversial. Photo-thermal ablation induces electronic excitation and thermalization whereas in photo-chemical ablation, covalent bonds in the polymer chains are directly broken by high-energy UV photons. Ablation mechanisms depend on laser characteristics such as wavelength, pulse duration, repetition rate, fluence and intensity, and on material properties such as absorption, reflectance and thermal conductivity. At the extreme intensities reached by ultrashort laser pulses absorption becomes nonlinear. Since the first report on laser ablation of polymers by Srinivasan and Kawamura in 1982 [36,37] a wide variety of polymers have been processed by using laser radiation with pulsewidth from the ns- to the fs-range and wavelengths in the UV, VIS and IR spectral regions aiming at explaining the nature of these mechanisms as well as incubation phenomena, studying the correlation of the laser processing parameters on the modification of the morphology, optical properties and chemical composition [38,39,40,41].

It is well known that the inscription of diffractive optical elements such as diffraction gratings can be used to modify the refractive index and hence the refractive power of an optical device by using short and ultrashort laser pulses with pulse energy below damage threshold [32,33,34,35,42,43]. In this work, we report on the fabrication of diffractive gratings in acrylic intra-ocular lenses by using high-repetition-rate ultrashort laser pulses. For this purpose, periodic linear patterns were inscribed on the surface and inside the polymer sample modifying the pulse energy, the scanning speed, and the inter-line spacing. Processed samples were analyzed by optical and contrast phase microscopy, SEM-EDX, confocal micro-Raman spectroscopy and diffractive techniques under illumination of a cw He-Ne laser.

## 2. Experimental

### 2.1. Laser Processing

As the laser source, a HighQ2-SHG femtosecond system (Spectra Physics, Santa Clara, CA, USA) delivering 250 fs laser pulses at a fixed wavelength of 520 nm and a repetition rate of 63 MHz was used to process the samples. The laser beam was focused on the surface and 150 µm beneath the surface by using a 100× long working distance infinity corrected microscope objective (NA = 0.6). According to the equation d_0_ = 2 × 1.22 × λ/NA [22], the theoretical diameter at the focal plane d_0_ was 2.1 µm. Pulse energy was set at 1 and 2 nJ with help of a calibrated neutral density filter. The sample, placed in a 3D motorized stage, was scanned to produce parallel tracks with lateral separation of 10 µm, 20 µm and 40 µm by using speeds of 0.25 mm/s, 0.50 mm/s and 1 mm/s. According to preliminary experiments, these values of pulse energy and scanning speed were considered the optimal to inscribe low damage diffraction gratings. As the substrate, a 320 µm thick hydrophobic UV-photo-reactive polybenzylmethacrylate polymer employed as ophthalmic intra-ocular lens was used (Contateq, Eindhoven, The Netherlands).

### 2.2. Characterization Techniques

Photographs were taken with a contrast phase microscope (B-800PH, Optika, Ponteranica, Italy) and a stereoscope lupe (SZM-LED2, Optika, Ponteranica, Italy). Optical transmission spectra were obtained by means of a spectrophotometer (U-3400, Hitachi, Abingdon, UK). Raman dispersion measurements were performed using a confocal Raman Spectrometer (Alpha 300 M+, WITec, Ulm, Germany) equipped with a thermoelectric-cooled CCD detector. A continuous wave 532 nm laser was used as the excitation source. The backscattered light was collected through a 20× microscope objective lens. The output power of the laser was kept below 20 mW in order to avoid significant local heating of the sample. A continuous 3 mW He-Ne laser at 632.8 nm was used to illuminate the diffraction gratings to characterize the diffractive modes and the refractive index variation.

## 3. Results

### 3.1. Ultrafast Laser Inscription of Diffraction Gratings

**Figure 1:**
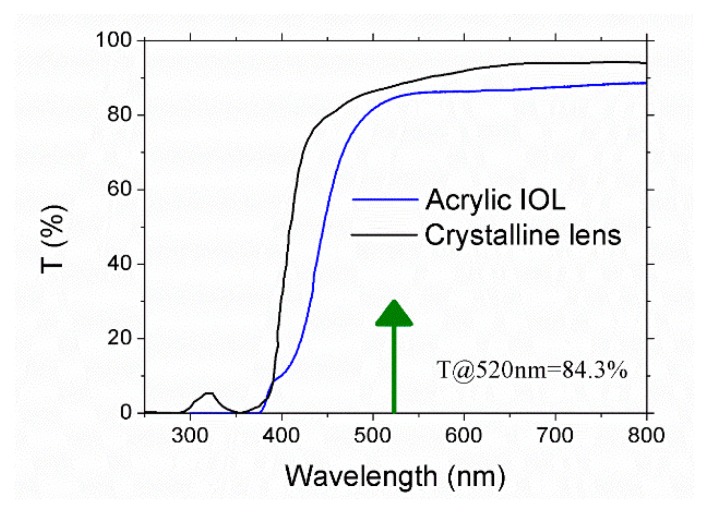
Optical transmission spectrum of acrylic intra-ocular lens. The arrow points out the laser wavelength used to process the sample, 520 nm, for which optical transmittance is 84.3%. Crystalline lens optical transmission spectrum is also included for comparison purposes.

Polymers used for ophthalmic applications commonly incorporate UV filters to mimic the natural characteristics of the optical tissue to be substituted. In particular, for the case of polymers used to replace the crystalline lens, these UV blocking chromophores prevent most UV radiation between 300 nm and 400 nm which is not useful for vision and may damage the retina [44]. Figure 1 shows the optical transmission spectrum of the acrylic IOL recorded at room temperature between 250 nm and 800 nm. It can be observed that the absorption cut-off wavelength is placed at 375 nm. In addition, optical transmittance at the laser wavelength used to process the samples, 520 nm is 84.3%. For comparison purposes the transmission spectrum of the crystalline lens is also included. 

**Figure 2:**
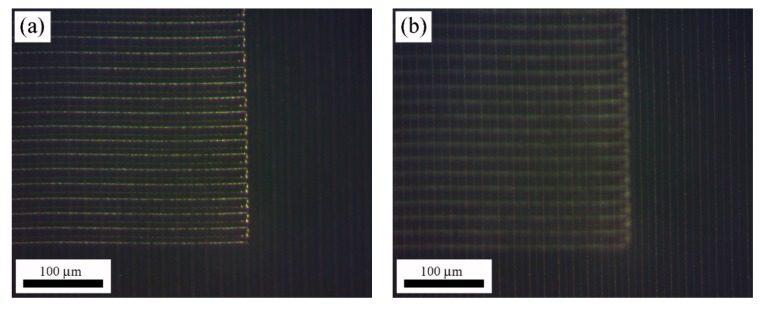
Image of periodic patterns inscribed on the surface (**a**) and 150 µm underneath the sample surface (**b**) at 1 mm/s, 10 µm inter-line spacing and 2 nJ pulse energy.

Since the optical absorption of the sample at the laser processing wavelength is very low only weak modification should be induced in the material at low processing rates, in the range of few µm/s. Nevertheless, the characteristics of this laser oscillator with 63 MHz repetition-rate and 250 fs pulse duration, properly combined with a high numerical aperture focusing objective, 0.6 NA, allow inducing non-linear absorption processes so that it was possible to produce linear diffraction gratings as on the surface as inside the material at high processing rates. In particular, diffraction gratings were processed on the surface and 150 µm underneath the surface at 0.25 mm/s, 0.5 mm/s and 1 mm/s with 10 µm, 20 µm and 40 µm inter-line spacing. It is worth highlighting that for ophthalmic applications diffraction gratings inscribed inside the sample in which the surface remains unaltered are preferred. Figure 2a shows a horizontal linear diffraction grating inscribed on the surface of the sample placed above a vertical linear diffraction grating inscribed 150 µm inside the material, Figure 2b. Both samples were processed at 1 mm/s with 10 µm inter-line spacing and 2 nJ pulse energy.

### 3.2. Microstructural and Compositional Characterization

**Figure 3:**
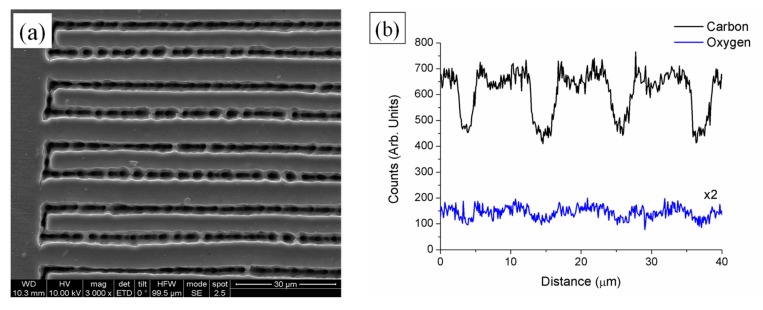
Top-view SEM micrograph of a periodic pattern inscribed on the surface of the acrylic IOL with 1 nJ pulse energy at 0.25 mm/s (**a**), and profile of semi-quantitative composition of carbon and oxygen content along the pattern measured by EDX analysis (**b**). Oxygen content has been doubled for clarification purposes.

Morphology and semi-quantitative chemical composition analyses of the processed IOL samples were carried out by SEM-EDX microanalysis to analyze the effects appeared in the polymer as a consequence of laser irradiation. Figure 3a shows a top-view micrograph of the processed area for a periodic pattern inscribed on the surface of the sample at 0.25 mm/s and 1 nJ pulse energy. As can be observed, linear tracks induced by the ultrashort laser radiation were quite inhomogeneous combining areas of low and high damage.

**Figure 4:**
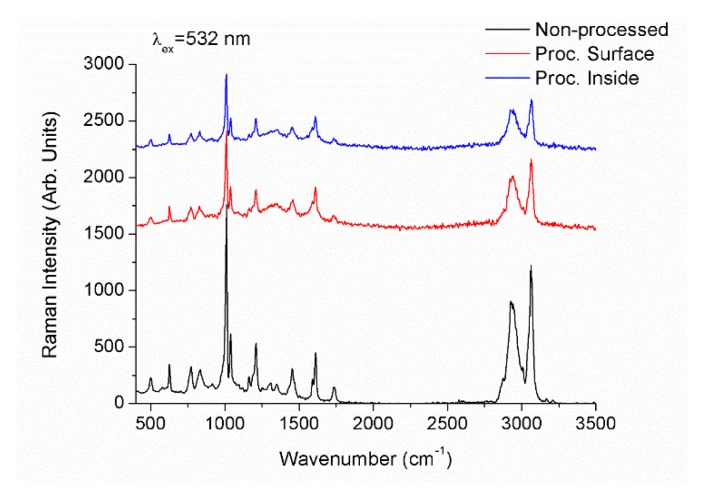
Micro-Raman spectra of non-processed acrylic IOL and processed areas on the surface and 150 µm underneath the surface.

It is worth mentioning that due to the high-repetition-rate of the laser source, the interaction with the polymer is produced in thermal regime. The repetition rate, or frequency, is a critical parameter in materials laser processing. Depending on the thermal properties of the material, accumulation of multiple laser pulses over the same point may result in an increase of the local temperature. The critical frequency, *f_cr_*, provides the cross-over between thermal and non-thermal regime and can be calculated according to [22]:

(1) $$f_{cr}=\frac{D_{th}}{d_{laser}^{2}},$$

where *D_th_* is the thermal diffusivity and *d_laser_* the laser beam diameter. Accounting that the thermal diffusivity of acrylic polymers ranges 10^−3^ cm^2^/s [45] and that the average beam diameter measured on the processed areas is 3 µm, the critical frequency results around 10 kHz. The frequency of the laser used to process these samples, 60 MHz, is well above this critical value so that the fabrication of diffraction gratings by using this laser source is in thermal regime. In these conditions it is expected the laser to induce thermal damage and decomposition. This assumption was confirmed by EDX analysis and micro-Raman spectroscopy. Figure 3b shows the profile of semi-quantitative compositional variation of both carbon and oxygen content in the periodic pattern inscribed on the surface of the acrylic IOL shown in Figure 3a. For clarification purposes, oxygen content has been doubled in the figure. Both carbon and oxygen content decreased along the irradiated area resulting in maximal variation at the center of the laser track. This diminution was approximately 30% and 40% for carbon and oxygen respectively. Figure 4 shows micro-Raman spectra of the non-processed IOL as well as the spectra acquired in periodic patterns processed as on the surface as 150 µm underneath the surface in the wavenumber region 400–3500 cm^−1^. The spectra show strong Raman bands at 498 cm^−1^, 626 cm^−1^, 773 cm^−1^, 831 cm^−1^, 1009.5 cm^−1^, 1037 cm^−1^, 1209.5 cm^−1^, 1310 cm^−1^, 1347 cm^−1^, 1611 cm^−1^ corresponding to ring vibrations and to alkyl groups. Bands at 1454 cm^−1^ and 1734 cm^−1^ can be ascribed to δ_a_(C-H) of O-CH_3_ and ν(C=O) of C-COO respectively. The broad band between 2600 cm^−1^ and 3200 cm^−1^ with maximal intensities at 2931 cm^−1^ and 3062 cm^−1^ arises from the convolution of symmetrical and asymmetrical stretching vibrations of CH and CH_3_ bonds. The observed Raman spectra agrees with those available in the literature [46,47,48,49]. Raman spectra of samples processed on the surface and inside the IOL showed a strong diminution of Raman intensity. Furthermore, bands placed at 1310 cm^−1^ and 1347 cm^−1^ turned into a broad band. Therefore, laser radiation induced a photo-thermal damage and hence the structure degradation of the acrylic intra-ocular lens.

### 3.3. Optical Characterization

**Figure 5:**
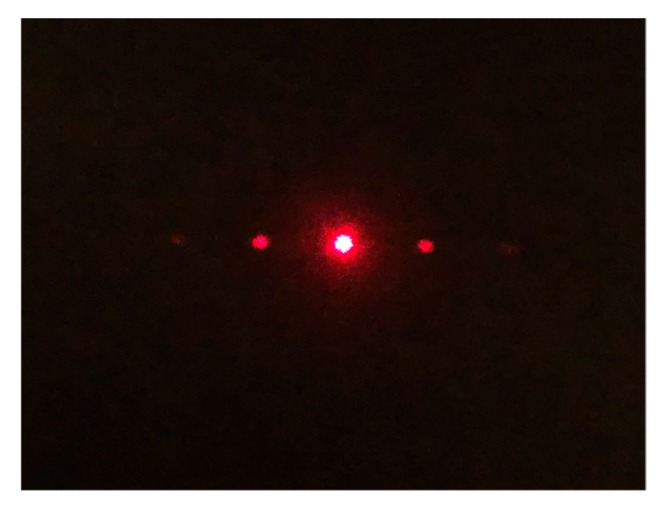
Far-field diffraction image of the output beam transmitted through the periodic structure processed 150 µm underneath the surface, with 20 µm inter-line spacing and 1 nJ pulse energy at 0.50 mm/s.

A continuous-wave He-Ne laser with emission at 632.8 nm was used to characterize the periodic patterns inscribed in the intra-ocular lenses. The angle of incidence of the He-Ne laser beam was set orthogonal to the samples. Only diffraction gratings inscribed inside the sample were optically characterized. All these samples showed diffraction patterns with diffraction angles according to the diffraction equation [42]. Intensities of zero and first diffracted orders were measured by using a power-meter to determine the 1st-order efficiency. As an example, Figure 5 shows the far-field diffraction image of the output beam transmitted through the periodic structure processed 150 µm underneath the surface, with 20 µm inter-line spacing and 1 nJ pulse energy at 0.50 mm/s.

**Figure 6:**
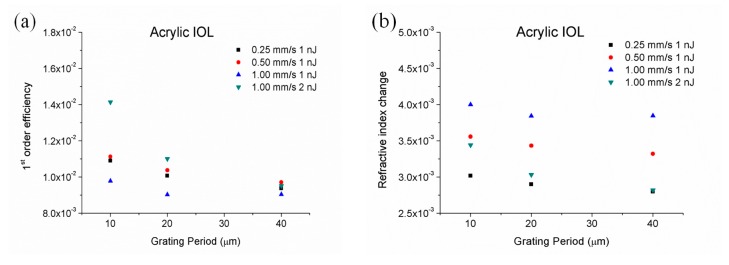
1st-order efficiency (**a**) and refractive index change (**b**) of diffraction gratings inscribed 150 µm underneath the surface of the acrylic IOL.

The magnitude of the refractive index modification, Δ*n*, was determined from the 1st-order efficiency according to the equation [50,51,52]:

(2) $$\Delta n=\frac{\lambda cos\;\theta tan\;h^{-1}\left(\sqrt{\eta }\right)}{\pi b},$$

where *λ* is the wavelength of the laser light used to assess the gratings (in our case, 633 nm), *θ* is the incident angle from the normal in the media (in our case, 0°), *η* is the 1st-order efficiency and *b* is the grating thickness. An optical microscope was used to measure the thickness of each grating in cross-section view, which were found to be 7 µm, 6 µm and 5 µm for 0.25 mms^−1^, 0.50 mms^−1^ and 1 mms^−1^ and 1 nJ respectively, and 7 µm for 1 mms^−1^ and 2 nJ. Figure 6 shows the 1st-order efficiency (a) and the refractive index change (b). It was observed that 1st-order efficiency decreased with the increase of the inter-line spacing whereas it increased as the energy delivered on the sample increased, either by increasing the pulse energy or by decreasing the scanning speed. It is worth highlighting that provided a pulse energy, its optimal value was achieved for a scanning speed of 0.50 mms^−1^. Concerning the refractive index change, it decreased with both the increase in the inter-line spacing and the energy delivered on the sample, with values ranging between 2.8 × 10^−3^ and 4.00 × 10^−3^. These values were similar to those reported in acrylate and silicone polymers [19,51,52,53,54].

## 4. Conclusions

Periodic patterns were successfully written on the surface and inside acrylic intra-ocular lenses using femtosecond laser pulses at high-repetition-rate. Patterns were assessed as a function of inter-line spacing, scanning speed and pulse energy. Compositional and microstructural characterization carried out by SEM-EDX and micro-Raman spectroscopy showed that laser radiation induced photo-thermal damage and decomposition. Optical characterization showed diffraction patterns under 632.8 nm cw-He-Ne laser illumination in all processed samples. First-order efficiency increased with the delivered energy on the sample and decreased with inter-line spacing, resulting in refractive index change between 2.8 × 10^−3^ and 4.00 × 10^−3^.

## References

[1] Rubinstein M., Colby R.H. (2003). Polymer Physics.

[2] Schnabel W. (2007). Polymers and Light.

[3] Hussain F., Hojjati M., Okamoto M., Gorga R.E. (2006). Polymer-matrix Nanocomposites, processing, manufacturing, and application: An overview. J. Compos. Mater..

[4] Jou J.H., Kumar S., Agrawal A., Li T.H., Sahoo S. (2015). Approaches for fabricating high efficiency organic light emitting diodes. J. Mater. Chem. C.

[5] Behl M., Razzaq M.Y., Lendlein A. (2010). Multifunctional shape-memory polymers. Adv. Mater..

[6] Kango S., Kalia S., Celli A., Njuguna J., Habibi Y., Kumar R. (2013). Surface modification of inorganic nanoparticles for development of organic-inorganic nanocomposites—A review. Prog. Polym. Sci..

[7] Scholz C. (2017). Polymers for Biomedicine: Synthesis, Characterization and Applications.

[8] Deligkaris K., Tadele T.S., Olthuis W., van den Berg A. (2010). Hydrogel devices for biomedical applications. Sens. Actuators B.

[9] Hunter A.C., Moghimi M.S. (2017). Smart polymers in drug delivery: A biological perspective. Polym. Chem..

[10] Calo E., Khutoryanskiy V.V. (2015). Biomedical applications of hydrogels: A review of patents and commercial products. Eur. Polym. J..

[11] Maulvi F.A., Soni T.G., Sha D.O. (2016). A review on therapeutic contact lenses for ocular drug delivery. Drug Deliv..

[12] Hassler C., Boretius T., Stieglitz T. (2011). Polymers for Neural Implants. J. Polym. Sci. Part B Polym. Phys..

[13] Allen N.S. (2010). Photochemistry and Photophysics of Polymeric Materials.

[14] Culbertson C.T., Mickleburgh T.G., Stewart-James S.A., Sellens K.A., Pressnall M. (2014). Micro total analysis systems: Fundamental advances and biological applications. Anal. Chem..

[15] Deepak K.L.N., Rao S.V., Rao D.N. (2010). Femtosecond laser-fabricated microstructures in bulk poly(methylmethacrylate) and poly(dimethylsiloxane) at 800 nm towards lab-on-a-chip applications. Pramana J. Phys..

[16] Ni M., Tong W.H., Choudhury D., Rahim N.A.A., Iliescu C., Yu H. (2009). Cell Culture on MEMS platforms: A review. Int. J. Mol. Sci..

[17] Suriano R., Kuznetsov A., Eaton S.M., Kiyan R., Cerullo G., Osellame R., Chichkov B.N., Levi M., Turri S. (2011). Femtosecond laser ablation of polymeric substrates for the fabrication of microfluidic channels. Appl. Surf. Sci..

[18] Narayana L., Kallepalli D., Soma V.R., Desai N.R. (2012). Femtosecond-laser direct writing in polymers and potential applications in microfluidics and memory devices. Opt. Eng..

[19] Kallepalli D.L.N., Desai N.R., Soma V.R. (2010). Fabrication and optical characterization of microstructures in poly(methylmethacrylate) and poly(dimethylsiloxane) using femto second pulses for photonic and microfluidic applications. Appl. Opt..

[20] Eaton S.M., de Marco C., Martinez-Vazquez R., Ramponi R., Turri S., Cerullo G., Osellame R. (2012). Femtosecond laser microstructuring for polymeric lab-on-chips. J. Biophotonics.

[21] Belluchi R. (2013). An introduction to intraocular lenses: Material, optics, haptics, design and aberration. Cataract.

[22] Misawa H., Juodkazis S. (2006). 3D Laser Microfabrication.

[23] Osellame R., Cerullo G., Ramponi R. (2012). Femtosecond Laser Micromachining, Photonic and Microfluidic Devices in Transparent Materials.

[24] Sola D., Escartin A., Cases R., Peña J.I. (2011). Crystal growth induced by Nd: YAG laser irradiation in patterning glass ceramic substrates with dots. Opt. Mater..

[25] Davis K.M., Miura K., Sugimoto N., Hirao K. (1996). Writing waveguides in glass with a femtosecond laser. Opt. Lett..

[26] Nolte S., Will M., Burghoff J., Tuennermann A. (2003). Femtosecond waveguide writing: A new avenue to three-dimensional integrated optics. Appl. Phys. A.

[27] Gamaly E.G., Juodkazis S., Misawa H., Luther-Davies B., Rode A.V., Hallo L., Nicolai P., Tikhonchuk V.T. (2008). Formation of nano-voids in transparent dielectrics by femtosecond laser. Curr. Appl. Phys..

[28] Sola D., Martinez de Mendibil J., Vazquez de Aldana J.R., Lifante G., Balda R., de Aza A.H., Pena P., Fernandez J. (2013). Stress-induced buried waveguides in the 0.8CaSiO_3_-0.2Ca_3_(PO_4_)_2_ eutectic glass doped with Nd^3+^ ions. Appl. Surf. Sci..

[29] Martinez de Mendivil J., Sola D., Vazquez de Aldana J.R., Lifante G., de Aza A.H., Pena P., Peña J.I. (2015). Ultrafast direct laser writing of cladding waveguides in the 0.8CaSiO_3_-0.2Ca_3_(PO_4_)_2_ eutectic glass doped with Nd^3+^ ions. J. Appl. Phys..

[30] Chen F., Vazquez de Aldana J.R. (2014). Optical waveguides in crystalline dielectric materials produced by femtosecond-laser micromachining. Laser Photonics Rev..

[31] Zoubir A., Lopez C., Richardson M., Richarson K. (2004). Femtosecond laser fabrication of tubular waveguides in poly (methyl methacrylate). Opt. Lett..

[32] Ding L., Blackwell R., Künzler J.F., Knox W.H. (2006). Large refractive index change in silicone-based and non-silicone-based hydrogel polymers induced by femtosecond laser micro-machining. Opt. Express.

[33] Ding L., Blackwell R.I., Künzler J.F., Knox W.H. (2008). Femtosecond laser micromachining of waveguides in silicone-based hydrogel polymers. Appl. Opt..

[34] Xu L., Knox W.H. (2011). Lateral gradient index microlenses written in ophthalmic hydrogel polymers by femtosecond laser machining. Opt. Mater. Express.

[35] Gandara-Montano G.A., Ivansky A., Savage D.E., Ellis J.D., Knox W.H. (2015). Femtosecond laser writing of freeform gradient index microlenses in hydrogel-based contact lenses. Opt. Mater. Express.

[36] Srinivasan R., Mayne-Banton V. (1982). Self-developing photoetching of poly(ethylene terephthalate) films by far-ultraviolet excimer laser radiation. Appl. Phys. Lett..

[37] Kawamura Y., Toyoda K., Namba S. (1982). Effective deep ultraviolet photoetching of polymethyl methacrylate by an excimer laser. Appl. Phys. Lett..

[38] Srinivasan V., Smrtic M.A., Badu S.V. (1986). Excimer laser etching of polymers. J. Appl. Phys..

[39] Srinivasan R., Braren B., Casey K.G. (1990). Nature of incubation pulses in the ultraviolet laser ablation of polymethyl methacrylate. J. Appl. Phys..

[40] Cain S.R., Burns F.C., Otis C.E. (1992). On single-photon ultraviolet ablation of polymeric materials. J. Appl. Phys..

[41] Blanchet G.B., Cotts P., Fincher C.R. (2000). Incubation: Subthreshold ablation of poly- (methyl methacrylate) and the nature of the decomposition pathways. J. Appl. Phys..

[42] Sola D., Lavieja C., Orera A., Clemente M.J. (2018). Direct laser interference patterning of ophthalmic polydimethylsiloxane (PDMS) polymers. Opt. Lasers Eng..

[43] Sola D., Alamri S., Lasagni A.F., Artal P. (2019). Fabrication and characterization of diffraction gratings in ophthalmic polymers by using UV direct laser interference patterning. Appl. Surf. Sci..

[44] Behar-Cohen F., Baillet G., de Ayguavives T., Ortega Garcia P., Krutmann J., Peña-García C., Reme C., Wolffsohn J.S. (2014). Ultraviolet damage to the eye revisited: Eye-sun protection factor (E-SPF^®^), a new ultraviolet protection label for eyewear. Clin. Ophthalmol..

[45] Rohde M., Hemberger F., Bauer T., Blumm J., Fend T., Häusler T., Hammerschmidt U., Hohenauer W., Jaenicke-Rössler K., Kaschnitz E. (2013). Intercomparison of thermal diffusivity measurements on CuCrZr and PMMA. High Temp. High Press..

[46] Willis H., Zichy V., Hendra P. (1969). The laser-Raman and infra-red spectra of poly (methyl methacrylate). Polymer.

[47] Pallikari F., Chondrokoukis G., Rebelakis M., Kotsalas Y. (2001). Raman spectroscopy: A technique for estimating extent of polymerization in PMMA. Mater. Res. Innov..

[48] Thomas K.J., Sheeba M., Nampoori V.P.N., Vallabhan C.P.G., Radhakrishnan P. (2008). Raman spectra of polymethyl methacrylate optical fibres excited by a 532 nm diode pumped solid state laser. J. Opt. Apure Appl. Opt..

[49] Rusciano G., Capaccio A., Pesce G., Sasso A. (2019). Experimental study of the mechanisms leading to the formation of glistenings in intraocular lenses by Raman spectroscopy. Biomed. Opt. Express.

[50] Mailis S., Anderson A.A., Barrington S.J., Brocklesby W.S., Greef R., Rutt H.N., Eason R.W., Vainos N.A., Grivas G. (1998). Photosensitivity of lead germanate glass waveguides grown by pulsed laser deposition. Opt. Lett..

[51] Park J.K., Cho S.H. (2011). Flexible gratings fabricated in polymeric plate using femtosecond laser irradiation. Opt. Lasers Eng..

[52] Scully P.J., Jones D., Jaroszynski D.A. (2003). Femtosecond laser irradiation of polymethylmethacrylate for refractive index gratings. J. Opt. A Pure Appl. Opt..

[53] Cho S.H., Chang W.S., Kim K.R., Hong J.W. (2009). Femtosecond laser embedded grating micromachining of flexible PDMS plates. Opt. Commun..

[54] Watanabe W., Matsuda K., Hirono S., Mochizuki H. (2012). Writing Speed Dependency of Femtosecond Laser Refractive Index Modification in Poly (dimethylsiloxane). J. Laser Micro Nanoeng..

